# Novel Human Gammapapillomavirus Species in a Nasal Swab

**DOI:** 10.1128/genomeA.00022-13

**Published:** 2013-03-07

**Authors:** Tung Gia Phan, Nguyen P. Vo, Matti Aronen, Laura Jartti, Tuomas Jartti, Eric Delwart

**Affiliations:** a Blood Systems Research Institute, San Francisco, California, USA; b Department of Laboratory Medicine, University of California, San Francisco, San Francisco, California, USA; c Pharmacology Department, School of Pharmacy, Ho Chi Minh City University of Medicine and Pharmacy, Ho Chi Minh City, Vietnam; d Department of Geriatrics, Turku City Hospital, Turku, Finland; e Department of Pediatrics, Turku University Hospital, Turku, Finland

## Abstract

A divergent human gammapapillomavirus (γ-HPV) genome in a nasal swab from an elderly Finnish patient with respiratory symptoms was genetically characterized. The L1 gene of HPV-Fin864 shared <70% nucleotide identity to other reported γ-HPV genomes, provisionally qualifying it as a new species in the *Gammapapillomavirus* genus.

## GENOME ANNOUNCEMENT

Members of the *Papillomaviridae* family are small double-stranded circular DNA viruses. Human papillomaviruses (HPVs) are highly diverse and have been classified into five genera, each made of multiple species that are further divided into types (1). A subset of HPVs cause anogenital or head and neck cancers (2–4), while others cause noncancerous skin growths. Other HPVs are commonly found on healthy human skin (5). Here, we describe a novel papillomavirus genome from a viral metagenomic analysis of a nasal swab from an elderly Finnish patient who was hospitalized due to a respiratory infection of unknown origin (negative for metapneumovirus, adenovirus, coronavirus, influenza virus, parainfluenza virus, respiratory syncytial virus, rhinovirus, and bocavirus 1) (6). A complete circular DNA viral genome was amplified using PCR and inverse PCR with primers designed from 454 pyrosequences showing significant BLASTn matches to HPVs. The circular genome (HPV-Fin864) was 7,247 bp, with a G+C content of 37%. Seven distinct open reading frames (ORFs) were identified on the same coding strand, including the early genes E6, E7, E1, E2, and E4 and the late genes L2 and L1. The long control region (LCR) between the L1 and E6 ORFs was 413 bp long, containing the TATA box (TATAAA) and four consensus E2-binding sites (ACC-AGAAGC-GGT [ACC-X6-GGT]) (7). Two characteristic zinc-binding domains (C-X2-C-X29-C-X2-C) separated by 37 amino acids were identified in E6 and one was identified in E7 (8). The E1 protein contained the nucleotide-binding domain of the helicase (GPPGTGKS [G-X4-GKT/S]) (9). The E1 protein contained a cyclin interaction RXL motif required for viral replication (10).

The L1 gene of HPV-Fin864 showed a best BLASTn match to HPV-156, a recently described γ-HPV species found in skin samples from immunocompetent patients (11). The *Gammapapillomavirus* genus currently consists of 10 viral species known to infect humans (1). Recently published genomes are expected to increase that number (11, 13). According to the International Committee on Taxonomy of Viruses (ICTV), the members of a papillomavirus species should share at least 70% nucleotide identity in the L1 gene (12). Pairwise nucleotide distance measurement showed that the L1 genes of HPV-156 and HPV-Fin864 shared 67% nucleotide identity. HPV-Fin864 therefore qualifies as a novel species in the *Gammapapillomavirus* genus, pending ICTV review. Given the association of human papillomaviruses with benign or malignant proliferative diseases of cutaneous and mucosal epithelia, the detection of HPV-Fin864 in the respiratory fluid of a patient with respiratory symptoms may have been coincidental.

### Nucleotide sequence accession number.

The complete genome sequence of HPV-Fin864 is available in GenBank under the accession no. KC311731.
